# Safety and Adequacy of Percutaneous Biopsies in Pediatric Orthotopic Kidney Transplantation

**Published:** 2012-02-01

**Authors:** F. E. Mackie, S. E. Kennedy, A. R. Rosenberg, G. Kainer, J. E. Frawley, K. S. Haghighi

**Affiliations:** 1 *Department of Transplant Surgery, Sydney Children’s Hospital Randwick and East Coast Renal Transplant Service, Sydney, Australia*; 2 *Department of Nephrology, Sydney Children’s Hospital Randwick and East Coast Renal Transplant Service, Sydney, Australia*; 3 *The University of New South Wales, Australia*

**Keywords:** Kidney transplantation, Biopsy, Safety, Pediatrics

## Abstract

Background: The gold standard for investigating the cause of renal graft dysfunction is renal biopsy. However, as this procedure is invasive and has inherent risks, its safety must be established.

Objective: To determine the safety of percutaneous renal biopsy in pediatric orthotopic renal transplantation.

Methods: Percutaneous renal biopsies performed on pediatric orthotopic renal transplants in a single center between 1987 and 2010 were studied. Biopsy specimen adequacy and post-procedure complications were reviewed by prospectively collected data.

Results: A total of 54 ultrasound “real-time” guided biopsies in 25 patients were performed. Minimum specimen adequacy was achieved in 98% of biopsy specimens. No major complications were identified; 6% of patients developed minor complications—*e.g.*, grade 3 macroscopic hematuria that did not require intervention.

Conclusion: Percutaneous renal biopsies using “real-time” ultrasound guidance on pediatric orthotopic kidney transplants is safe.

## INTRODUCTION

Kidney transplantation is the optimal treatment for children with end-stage renal disease. However, transplantation of adult sized kidneys into young children is technically challenging due to the small size of recipients and relative size mismatch of donor and recipient blood vessels. Transperitoneal transplantation has been developed to overcome some of these difficulties; it results in orthotopic placement of the graft into the retroperitoneal space as opposed to the traditional iliac fossa and allows anastomosis of donor vessels to the recipient aorta and inferior vena cava. One possible shortcoming of this technique is that biopsy of the graft becomes more difficult, and can be associated with risks of bleeding, morbidity, loss of graft and even mortality. To date, there is limited data on the safety and adequacy of biopsy in orthotopically located grafts. In this retrospective analysis of a prospective database, we describe the safety profile and specimen adequacy of biopsies of pediatric orthotopic kidney transplants at a single center.

## PATIENTS AND METHODS

All pediatric orthotopic renal transplants performed at Sydney Children’s Hospital, Randwick (previously known as The Prince of Wales Children’s Hospital) between January 1987 and May 2010 were identified. Patients on whom percutaneous renal biopsies had been performed since 1998, when data regarding biopsy indication, specimen adequacy and complications was prospectively collected, were identified and included in this study.

Patient details were extracted from the medical records. Biopsy specimen adequacy was graded according to glomeruli number as per the Banff 97 biopsy criteria [1] with a “minimal sample” containing seven glomeruli and an “adequate specimen” containing 10 or more glomeruli. Complications were categorized as “minor” or “major,” with minor complications including macroscopic hematuria, hematoma and increased length of hospital stay. Major complications were hemorrhage requiring blood transfusion or surgical intervention, urine collections or incidents requiring surgical or radiological intervention, loss of graft and patient death.

Percutaneous biopsies were initially performed as inpatient procedures with hospital admission for 24 hours of observation. In recent years, however, biopsies have been performed as a day procedure. All biopsies were performed by a pediatric nephrologist or experienced nephrology fellow using “real-time” ultrasound guidance, assisted by a radiologist. Biopsies were performed under general anesthesia or sedation with additional local anesthesia. A posterior lumbar approach was used with the patient in the prone position. Body curvature was achieved by placing a towel for support under the abdomen whilst prone. The biopsy was usually taken from the lower pole of the kidney. However, in some cases this was inaccessible as it lay beneath the iliac crest. In such instances, the upper pole was biopsied, except if the kidney was right sided when the risk of liver injury was considered to be too great. At least two tissue cores were obtained via an 18-G needle on a spring loaded automated biopsy gun.

Following the biopsy, an ultrasound was immediately performed by the radiologist for identification of hematoma formation.

Post-procedure, patients were confined to bed rest and observations included regular blood pressure, pulse rate and temperature. The biopsy site was monitored for bleeding or hematoma formation. All urine passed was monitored for macroscopic and microscopic hematuria by urinary dipstick. For the purposes of this study, bleeding was classified into three grades as per Vidhun, *et al* [2].

## RESULTS

Thirty-six orthotopic kidney transplants (16 females) were performed between 1987 and 2010. The median age at transplantation was 6 (range: 1–13) years. Twenty-seven children weighed less than 25 kg (weight range: 11–47 kg). Fifty-four percutaneous biopsies were performed on 25 patients. The median age at time of biopsy was 8 (range: 2–17) years. The median time elapsed from transplantation was three years (range: 5 days to 14 years). The indications for biopsy are shown in Table 1.

**Table 1:** Indications for percutaneous renal biopsy

**Indication for biopsy**	**Number of cases (%). Total = 54**
Deterioration of renal function	47 (87%)
Protocol	5 (9%)
Proteinuria	1 (2%)
Delayed graft function	1 (2%)

Adequacy of biopsy specimens was determined according to the Banff 97 criteria. Forty-nine (91%) specimens included more than 10 glomeruli and 53 (98%) included more than seven glomeruli.

Three (6%) patients developed macroscopic hematuria (grade 3) within four hours of biopsy. Macroscopic hematuria spontaneously resolved by 36 hours in two and by 48 hours in the other. No patient’s hospital discharge was delayed by greater than 24 hours. There were no episodes of macroscopic hematuria after discharge. No patient required subsequent blood transfusion. There were no major complications such as infection, incident requiring surgical or radiological intervention, loss of graft or patient death in this series of patients.

## DISCUSSION

Biopsies of kidney transplants may provide important information to guide clinical management. It is critical that the risk of complications and the ability to attain an adequate specimen is balanced against possible beneficial diagnostic information. Our data demonstrated a biopsy specimen adequacy rate of 98% and complications confined to transient (24–48 hours post-biopsy) grade 3 hematuria in three patients (6%).

Percutaneous biopsies of orthotopically located grafts have been reported to be associated with increased risk of bowel perforation or vessel injury [2]. Caution must be taken as the relatively large size of adult kidneys in the orthotopic position may make access to the upper or lower pole more difficult. Our results suggest that with the use of correct patient positioning, an 18-G needle and ultrasound guidance, the risk of major complications is low and as previously reported [2], is comparable to that described in heterotopic transplants [3-5]. We did not identify any significant perinephric hematoma formation following biopsy. Vidhun, *et al*, identified an incidence of 13.4% of perinephric hematoma formation most of which were less than 1 cm in size. These were found to be more common in biopsies performed with a larger 16-G needle, as opposed to the 18-G needle used in our study, but did not result in requiring any subsequent blood transfusion or intervention [2]. In fact, our results are even superior to percutaneous native kidney biopsies [5]. Other complications which have been reported in biopsies of iliac fossa grafts include a 7.6% incidence of arteriovenous fistula formation [6]. This complication was not formally examined in our study. However, biannual ultrasound is routinely performed in our transplant recipients and no fistulae have been observed. The reported rates of major complications in biopsies of iliac fossa grafts requiring blood transfusion has varied widely from 0.5% to 9% [2-5,7]. Consequently, larger studies would be required to determine true incidence rates. 

There are no consensus upon guidelines on the most appropriate in-hospital observation period following biopsy. Our study did not find an increased complication rate following transition from hospital inpatient admission for biopsy to outpatient biopsy. Other authors have suggested an observation period of either 6 or 12 hours [7,8]. In our study, all cases of macroscopic hematuria were evident by four hours after biopsy suggesting that six to eight hours of observation may be enough. However, this would depend on factors such as the patient’s ability to promptly access acute care facilities. 

Importantly, to our knowledge this is the first study to selectively describe the adequacy of specimens attained by percutaneous biopsy of transperitoneal transplants. Our specimen adequacy was comparable to heterotopic renal transplant biopsy studies [3] despite using the smaller 18-G needle, and would be expected to provide information required to guide post-transplant management. We have also identified comparable data on biopsy complications, and based on our findings, we would suggest that renal biopsies of orthotopic kidney transplants can be performed safely under ultrasound guidance with minimal complications.

## Notes

Financial Support: **None.**
